# A robust prognostic gene expression signature for early stage lung adenocarcinoma

**DOI:** 10.1186/s40364-016-0058-3

**Published:** 2016-02-19

**Authors:** Marcin Krzystanek, Judit Moldvay, David Szüts, Zoltan Szallasi, Aron Charles Eklund

**Affiliations:** Department of Systems Biology, Center for Biological Sequence Analysis, Technical University of Denmark, Lyngby, Denmark; Department of Tumor Biology, National Koranyi Institute – Semmelweis University, Budapest, Hungary; Institute of Enzymology, Research Centre for Natural Sciences, Hungarian Academy of Sciences, Budapest, Hungary; Computational Health Informatics Program (CHIP), Boston Children’s Hospital, Boston, USA; Harvard Medical School, Boston, USA

**Keywords:** Lung adenocarcinoma, Prognostic biomarker, Gene expression, Early stage cancer

## Abstract

**Background:**

Stage I lung adenocarcinoma is usually not treated with adjuvant chemotherapy; however, around half of these patients do not survive 5 years. Therefore, a reliable prognostic biomarker for early stage patients would be critical to identify those most likely to benefit from early additional treatments. Several studies have searched for gene expression prognostic biomarkers for lung adenocarcinoma, but these have not yielded a widely accepted prognosticator.

**Results:**

We analyzed gene expression from seven published lung adenocarcinoma cohorts for which we included only stage I and II patients who were not given adjuvant therapy. Seven genes consistently obtained statistical significance in Cox regression for overall survival. The combined signature has a weighted mean hazard ratio of 3.2 in all cohorts and 3.0 (C.I. 1.3–7.4, p < 0.01) in an independent validation cohort and is strongly correlated with previously published signatures of chromosomal instability and cell cycle progression.

**Conclusions:**

The new prognostic signature, if validated prospectively, may enable better stratification and treatment of early stage lung cancer patients.

**Electronic supplementary material:**

The online version of this article (doi:10.1186/s40364-016-0058-3) contains supplementary material, which is available to authorized users.

## Background

Lung cancer has the third highest incidence rate and the highest mortality rate of all cancer types. For non-small cell lung cancer (NSCLC), the 5-year survival rate remains below 15 % [1, 2]. Given the difficulties with treatment of advanced NSCLC, the most promising possibility of improving outcomes may be efficient diagnosis and treatment of early stage cases. One of the most important clinical decisions in these patients is whether to give adjuvant chemotherapy in addition to surgical resection. At present postoperative chemotherapy is not recommended for patients with completely resected stage IA NSCLC with 1A level of evidence, and can be considered in stage IB disease and a primary tumor >4 cm with 2B level of evidence [2]. Nonetheless, only up to 73 % of stage IA and 58 % of stage IB patients survive 5 years [1]. Therefore, identification of patients who are likely to benefit from adjuvant treatment – even with NSCLC of stage I – would be of strong diagnostic and prognostic relevance.

A similar problem has been extensively studied and to a significant extent answered in node negative estrogen receptor positive breast cancer. A gene expression signature was obtained that identifies patients with high risk of recurrence who benefit from additional chemotherapy [3]. Identification of such a gene expression signature often initiates with the quantification of a large number of genes on a patient cohort where outcome is known and then the most informative genes are selected and further validated.

Similar strategies have been applied to lung adenocarcinoma as well. Several cohorts containing early to mid stage lung cancer patients have been subjected to transcriptomic analysis, mainly by microarray [4–10]. However, no reliable, consistent gene expression based prognosticator has emerged from these efforts. One possible reason for this failure could be that some of these studies followed a suboptimal strategy in one or more possible ways: 1) searching for a general NSCLC prognosticator, as opposed to lung adenocarcinoma (LUAD)- or lung squamous carcinoma (LUSC)-specific signatures [4, 5], 2) doing the analysis in a treated/untreated mixed population [4, 6, 9], 3) ignoring the possibility of technical bias in the microarray data [11, 12]. On the other hand, a PCR-based gene expression signature of cell cycle progression (CCP), which was *not* derived from lung cancer patients, was found to be prognostic in three early stage LUAD cohorts and validated in a PCR-based study [13, 14].

The increasing availability of lung cancer datasets makes it possible to look for a robust signature comprising of genes that would not otherwise be found if the study had been conducted on only a single dataset. Therefore, we set out to perform a meta-analysis of several lung cancer microarray datasets to see if we could identify a gene expression signature that is prognostic in early stage lung adenocarcinoma patients who were not given chemotherapy.

## Methods

Microarray data were downloaded from the GEO database (GSE8894, GSE14814, GSE30219, GSE31210, GSE37745, GSE50081), except Shedden et al., which was downloaded from caarraydb.nci.nih.gov (Table 1). All calculations were performed using the R programming language. Each gene expression dataset was normalized with the RMA algorithm except GSE9984, for which we were unable to obtain the original raw data, and therefore we used the version downloaded directly from GEO which had been normalized with the GCRMA algorithm [4]. Each dataset, except GSE9984, was also corrected for two sources of potential bias: the RNA degradation captured as the average decrease in expression between 5’ probes and 3’ probes (degradation bias metric) and the diversity of starting mRNA (RMA IQR bias metric) that remained after RMA normalization. This step was performed using “bias” package version 0.0.5 [11]. Weighted average of hazard ratios was calculated as a mean weighted by size of each dataset.

**Table 1:** Number of patients in the cohorts included in this study

		Included^a^		
Cohort	Available	Stage I	Stage II	Total
Lee et al. 2008 [4]	138	39	12	51
Zhu et al. 2010 [5]	133	20	12	32
Shedden et al. 2008 [6]	505	164	43	207
Rousseaux et al. 2013 [7]	307	81	3	84
Okayama et al. 2012 [8]	246	162	42	204
Botling et al. 2013 [9]	196	31	4	35
Der et al. 2014 [10]	181	92	35	127

^a^passed inclusion criteria: adenocarcinoma, no chemotherapy

Gene expression and clinical covariates from the validation cohort were downloaded from the TCGA Data Portal using Data Matrix (https://tcga-data.nci.nih.gov/tcga/dataAccessMatrix.htm), selecting all available tumors on 15^th^ Jan 2016, disease: LUAD and data type: RNASeqV2. Expression values were extracted from files containing RSEM gene-normalized results and we normalized them across samples using the average gene expression of each tumor [15, 16].

## Results

We performed a literature and database search and identified seven publicly available gene expression data sets with overall or recurrence-free survival data and with at least 30 patients meeting the following criteria: 1) profiled on the Affymetrix HG-U133A or HG-U133 Plus 2.0 platform; 2) adenocarcinoma subtype by histological report; 3) pathological stage I or II; and 4) not given neoadjuvant, adjuvant, or targeted therapy (Table 1). Each data set (except Lee et al. [4]) was normalized individually and adjusted to reduce technical bias [11].

To identify genes whose expression level is prognostic, we applied the following procedure to each of the 22,277 common probe sets. We split each cohort into two groups according to the expression value of the probe set, and applied Cox proportional hazards regression and a log-rank test of statistical significance on these two groups. If the hazard ratio had the same directionality in all cohorts, and the P value was below 0.05 in any six of the seven cohorts, the probe set was considered prognostic. We expect this procedure to yield an individual type I error rate of 1.0 × 10^−7^, and with Bonferroni correction for 22,277 probe sets, a family-wise error rate of 0.00036. This procedure resulted in seven probe sets, each representing a unique gene (Table 2).

**Table 2:** Hazard ratios for the individual ESLA-7 genes in each cohort

Gene	ADAM10		DLGAP5		RAD51AP1		FGFR10P		NCGAP		KIF15		ASPM	
Probe set	202604_x_at		203764_at		204146_at		205588_s_at		218663_at		219306_at		219918_s_at	
Cohort	HR	p	HR	p	HR	p	HR	p	HR	p	HR	p	HR	p
Lee et al. 2008 [4]	3.0	**	2.3	*	4	***	2.9	**	2.3	*	2.5	*	2.5	*
Zhu et al. 2010 [5]	0.7		3.1	*	3.7	*	3.2	*	4.7	**	3.9	*	4.1	**
Shedden et al. 2008 [6]	1.7	*	1.7	*	1.7	*	1.6	*	1.8	*	1.6	*	1.7	*
Rousseaux et al. 2013 [7]	2.6	**	2.4	**	2.7	**	1.9	*	2.3	**	2.3	**	1.9	*
Okayama et al. 2012 [8]	4.3	***	2.9	**	2.9	**	3.4	**	2.4	*	2.8	*	4.2	***
Botling et al. 2013 [9]	2.5	*	1.7		1.0		1.3		1.3		1.7		1.1	
Der et al. 2014 [10]	2.2	**	2.5	**	2	*	2.1	**	2.5	**	2.4	**	2	*

*p ≤ 0.05, **p ≤ 0.01, ***p ≤ 0.001

**Fig. 1:**
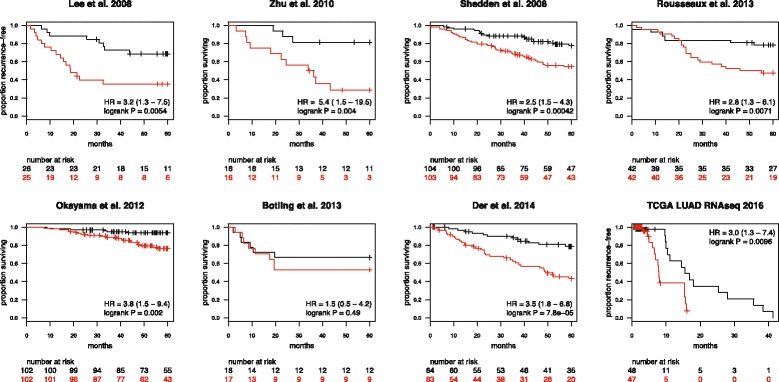
Kaplan-Meier overall or recurrence-free survival estimates for seven early stage lung adenocarcinoma cohorts and the TCGA LUAD validation cohort [4–10, 16]. Each cohort was split according to ESLA-7 score (red: > median, black: ≤ median)

The expression values of the seven probe sets were strongly positively correlated, so we defined a prognostic score of an individual tumor as the mean log_2_ expression value of the seven probe sets (termed “ESLA-7”, for early stage lung adenocarcinoma). We found that the ESLA-7 score, when used to stratify each cohort into two groups of equal size, is prognostic in six of the seven individual cohorts (Table 2 and Fig. 1). Notably, the ESLA-7 score was more prognostic than any of the individual probe sets. Overall, the weighted average hazard ratio of ESLA-7 was 3.2, or 3.6 if the Botling cohort was omitted (Fig. 1).

We next performed a multivariate analysis, adjusting for stage, age and stratifying for gender. No other clinical data types were available in all cohorts. In this analysis, ESLA-7 showed a HR ranging between 1.1–4.9 in the individual cohorts, with a weighted mean of 2.7 and statistical significance in all cohorts except Botling et al. 2008 (Table 3).

**Table 3:** Performance of ESLA-7 in multivariate setting when adjusted for age, stage and stratified for gender

Cohort	HR	CI	p	N	events
Lee et al. 2008 [4]	2.9	1.2–7.1	*	51	24
Zhu et al. 2010 [5]	4.9	1.3–18.1	*	32	14
Shedden et al. 2008 [6]	2.0	1.2–3.5	*	207	64
Rousseaux et al. 2013 [7]	2.6	1.2–5.8	*	84	31
Okayama et al. 2012 [8]	3.1	1.2–7.9	*	204	27
Botling et al. 2013 [9]	1.1	0.3–3.9		35	14
Der et al. 2014 [10]	3.2	1.6–6.2	***	127	44

*p ≤ 0.05, **p ≤ 0.01, ***p ≤ 0.001

To assess the performance of ESLA-7 in an independent cohort that was not used to derive the signature, we analyzed RNA-seq gene expression data from the TCGA LUAD cohort. To reduce the effects of varying chemotherapy regimes in the TCGA cohort, we analyzed recurrence-free survival (RFS) in stage I and II patients from this cohort. The majority of early stage LUAD patients do not receive chemotherapy until recurrence; therefore this analysis could be considered as a more accurate assessment of true prognostic performance. Here, ESLA-7 was statistically significant (HR = 1.8, C.I. 1.3–2.6, p < 0.001, Additional file 1: Figure S1A). Subsequently, we used available treatment information to censor the survival times at the time of initiation of chemotherapy, thus creating a subcohort of patients who did not receive chemotherapy during the followup period. RFS analysis of stage I and II, untreated patients (N = 95) showed HR = 3.0 (C.I. 1.3–7.4, p < 0.01, Figure 1 and Additional file 1: Figure S1B).

**Fig. 2:**
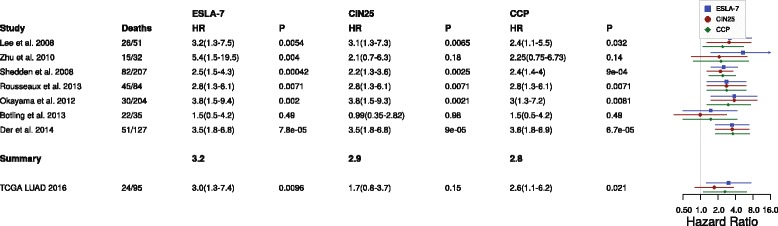
Forest plot indicating the performance of the ESLA-7, CIN25 [17] and CPP [13] signatures in the seven early stage lung adenocarcinoma cohorts and in the TCGA LUAD validation cohort

Since most of the seven genes were annotated with functions related to chromosomal instability (CIN), we asked whether we could achieve similar prognostic performance with our previously described CIN25 chromosomal instability signature or with the previously published cell cycle progression (CCP) signature [13, 14, 17]. We applied the CIN25 and the CCP signatures in the same way as the ESLA-7 signature and found that ESLA-7 on average performed better than both CIN25 and CCP (weighted mean hazard ratio of 3.2, 2.9 and 2.8 respectively, Fig. 2). CIN25 and CCP scores also showed trends similar to ESLA-7 when using TCGA LUAD RNAseq data (Additional file 1: Figure S1C-F). Additionally, the correlation between ESLA-7 and CIN25 within each cohort was very high, ranging from 0.88 to 0.98, suggesting that ESLA-7 to a large extent quantifies CIN. Similarly correlation between ESLA-7 and CCP ranged from 0.89 to 0.99.

**Fig. 3:**
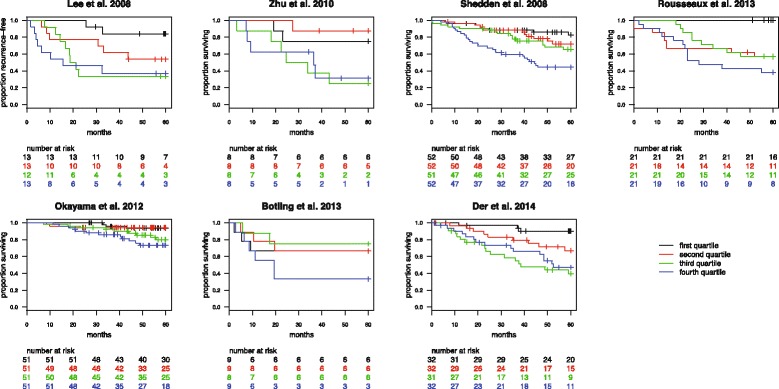
Kaplan-Meier survival estimates for the seven early stage lung adenocarcinoma cohorts, split according to ESLA-7 score quartiles

To assess the performance of ESLA-7 without arbitrary stratifying the cohorts into two equally sized groups, we performed Cox regression using ESLA-7 as a continuous variable. Association with overall survival (or recurrence-free survival in the Lee et al. 2008 cohort) was statistically significant in all cohorts except Botling et al. 2013 (weighted mean 2.1, Table 4). Additionally, we explored whether the ESLA-7 score might provide further prognostic stratification into more than two groups, using the ESLA-7 score to stratify each cohort into four groups of equal size. For cohorts with sufficient number of patients in each stratum a clear trend was apparent (Fig. 3). Patients in the first quartile had better survival than patients in the other three quartiles, and, especially, patients in the fourth quartile. No such trend was apparent in the Zhu et al. 2010 [5] and Botling et al. 2013 [9] cohorts, possibly due to the smaller numbers of patients in these two cohorts.

**Table 4:** Hazard ratios of cox regression using ESLA-7 as continuous variable

Cohort	HR	CI	p	N	events
Lee et al. 2008 [4]	1.8	1.3–2.6	***	51	24
Zhu et al. 2010 [5]	3.8	1.1–12.1	*	32	14
Shedden et al. 2008 [6]	2.4	1.6–3.5	***	207	64
Rousseaux et al. 2013 [7]	2.2	1.5–3.3	***	84	31
Okayama et al. 2012 [8]	1.9	1.3–2.8	***	204	27
Botling et al. 2013 [9]	1.2	0.7–2.2		35	14
Der et al. 2014 [10]	1.6	1.1–2.2	*	127	44

*p ≤ 0.05, **p ≤ 0.01, ***p ≤ 0.001

Finally, we applied ESLA-7 to five lung squamous cell carcinoma (LUSC) cohorts [4, 5, 7, 9]. Median split according to ESLA-7 value did not yield statistical significance in any of the LUSC cohorts (Additional file 2: Table S1 and Additional file 3 ). We attempted to create a separate signature for lung squamous cell carcinoma (LUSC). However, similar methodology applied to LUSC patients did not yield a robust expression signature (data not shown).

## Discussion

We used simple methodology to derive a robust prognostic gene expression signature for early stage lung adenocarcinoma. The signature was validated in an independent cohort, however, with 95 patients in the regression, of which many left the study or were censored due to the methodology within the first semester of the follow-up, one should treat this result with caution. If successfully validated in an independent clinical trial, the ESLA-7 signature could potentially be used for guiding clinical onocologist decisions on whether an individual early stage lung adenocarcinoma patient, especially a patient with stage I disease, should receive chemotherapy after surgical resection of the tumor. The seven genes could be combined with an additional small panel of reference genes, as has been done in similar prognostic signatures for other cancer types [3, 14, 18].

High correlation among ESLA-7 and the two other signatures suggests that to a large degree they may be quantifying the same biological processes. CIN25 was developed as a signature of chromosomal instability from specific genes whose expression was consistently correlated with aneuploidy in several types of tumors. As with CIN25, net overexpression of ESLA-7 was predictive of poor clinical outcome. Both signatures contain genes with function connected directly with kinetochore assembly: KIF20A, KIF4A, TPX2, PRC1, and TTK in CIN25; and KIF15, DLGAP5, and ASPM in ESLA-7. Also, both contain genes that are part of condensin complexes: NCAPG in ESLA-7; and NCAPD2 in CIN25. Both kinetochores and condensin complexes play central roles in chromosome assembly and segregation. Overall, both signatures point towards chromosomal instability as an important factor in early stage patient outcome.

Only a single gene, RAD51-associated protein 1 (RAD51AP1), is present in both signatures. This gene plays an important role in homologous recombination-mediated chromosome damage repair by enhancing the recombinase activity of RAD51.

The ESLA-7 signature includes genes ADAM10 and FGFR10P, which have been reported to contribute to oncogenesis of various cancer types including lung cancer, and whose function indicates that they may take part in oncogenic transformation and/or increase cell proliferation by interaction with major signaling cascades (ERK1/2, p38 MAPK, and STAT, Notch1, RAS/MAPK, PLC-γ, and PI3K/AKT). The slightly better performance of ESLA-7 as compared to CCP might be due to additional information carried by ADAM10 and/or FGFR10P, which are slightly less correlated with the remainder of the ESLA-7 genes (Additional file 4: Figure S2).

Interestingly, forkhead box MI (FOXM1), which takes part in the regulation of spindle assembly genes, nearly passed our criteria. This gene is also one of the top CIN25 and CCP genes and, more recently, was identified as predictor of adverse outcomes in various malignancies in a cross-platform, cross-cancer study [19].

While we found a monotonic correlation between the CIN signature and prognosis in LUAD, we previously found a non-monotonic relationship between CIN and prognosis in LUSC, in which very low and very high levels of CIN result in increased survival compared to the rest of the cohort [20].

## Conclusions

If CIN is indeed highly prognostic in early stage lung cancer, as our results indicate, then this would suggest that LUAD and LUSC cohorts should be analyzed separately. The new prognostic signature, if further validated prospectively, will enable better stratification and reduce overtreatment of early stage lung cancer patients.

### Statement on ethics approval

In this study we used publicly available data collected with patients consent approved by relevant institutional review board following declaration of Helsinki.

Data was approved by following institutional boards: Institutional review board of Samsung Medical Center, and written informed consent was obtained (IRB 2005-12-034), The University Health Network Research Ethics Board, Institutional Review Board of each of the four institutions’ (University of Michigan Cancer Center (UM), Moffitt Cancer Center (HLM), Memorial Sloan-Kettering Cancer Center (MSK) and the Dana-Farber Cancer Institute (CAN/DF)), Institutional Review Board of the National Cancer Center, Tokyo, Japan, Uppsala regional ethical review board, reference #2006/ 325 and Linkoeping regional ethical review board, reference #2010/44-31, Research Ethics Board of University Health Network (UHN181), for TCGA data used in this study declaration on ethics is as follows: All specimens were obtained from patients with appropriate consent from the relevant institutional review board.

## Additional files

Additional file 1: Figure S1A. TCGA LUAD RNAseq − stage I and II ESLA − 7, DFS. **Figure S1B**: TCGA LUAD RNAseq − stage I and II ESLA − 7, DFS − no treatment. **Figure S1C**: TCGA LUAD RNAseq − stage I and II CIN25, DFS. **Figure S1D**: TCGA LUAD RNAseq − stage I and II CIN25, DFS − no treatment. **Figure S1E**: TCGA LUAD RNAseq − stage I and II CCP, DFS. **Figure S1F**: TCGA LUAD RNAseq − stage I and II CCP, DFS − no treatment. (PDF 20 kb) Additional file 2: Table S1. Performance of ESLA-7 in chosen lung squamous datasets. Median split according to ESLA-7 score. *p ≤ 0.05, **p ≤ 0.01, ***p ≤ 0.001. (DOCX 59 kb) Additional file 3:
**TCGA LUSC clinical data.** (TXT 124 kb) Additional file 4: Figure S2. GSE8894 NSCLC Adeno untreated. (PDF 11 kb)

## Abbreviations

CCP: cell cycle progression (signature)
CIN: chromosomal instability (signature)
ESLA: early stage lung adenocarcinoma (signature)
GEO: gene expression omnibus
LUAD: lung adenocarcinoma
LUSC: lung squamous carcinoma
NSCLC: non small lung cancer
OS: overall survival
PCR: polymerase chain reaction
RFS: recurrence-free survival
