# Supplementary material for: A robust prognostic gene expression signature for early stage lung adenocarcinoma
Source: Biomark Res. 2016 Feb 19;4:4. doi: 10.1186/s40364-016-0058-3 (PMC4761211; doi:10.1186/s40364-016-0058-3)

Supplementary Figure 1A
TCGA LUAD RNAseq − stage I and II
ESLA−7, DFS

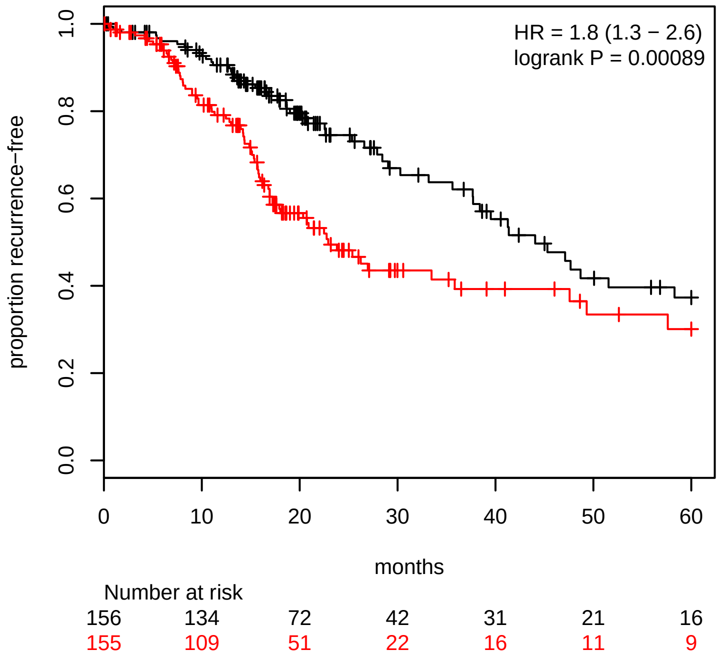

Supplementary Figure 1B
TCGA LUAD RNAseq − stage I and II
ESLA−7, DFS − no treatment

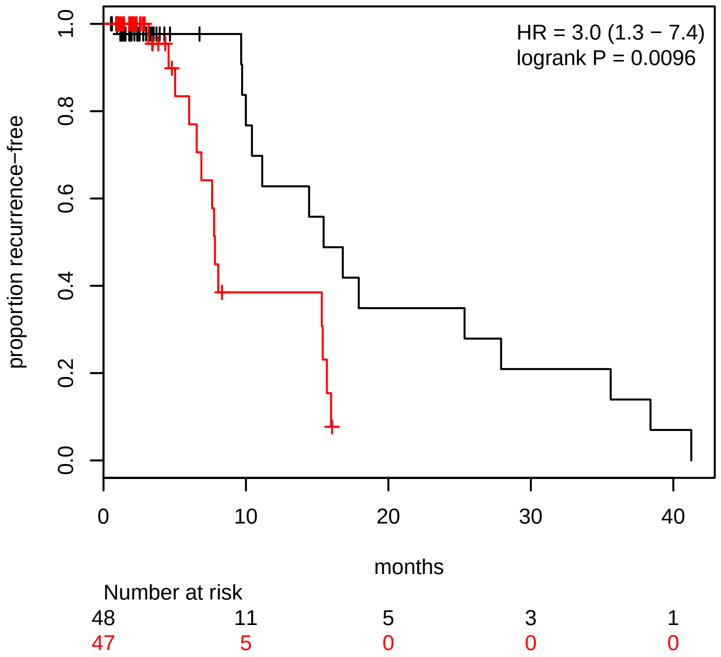

Supplementary Figure 1C
TCGA LUAD RNAseq − stage I and II
CIN25, DFS

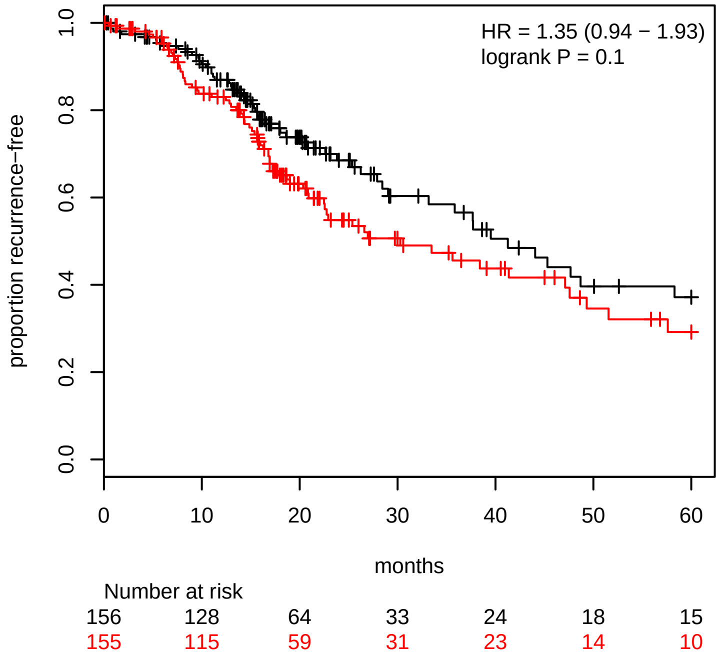

Supplementary Figure 1D
TCGA LUAD RNAseq − stage I and II
CIN25, DFS − no treatment

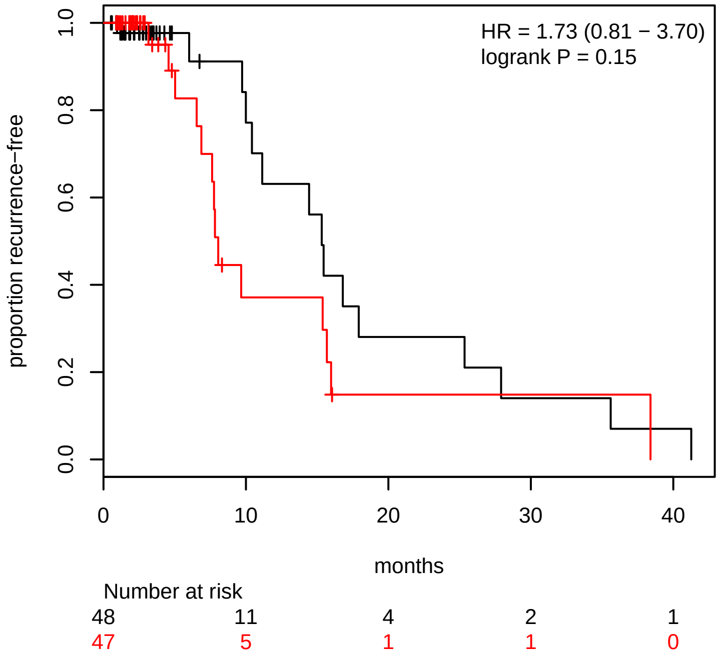

Supplementary Figure 1E
TCGA LUAD RNAseq − stage I and II
CCP, DFS

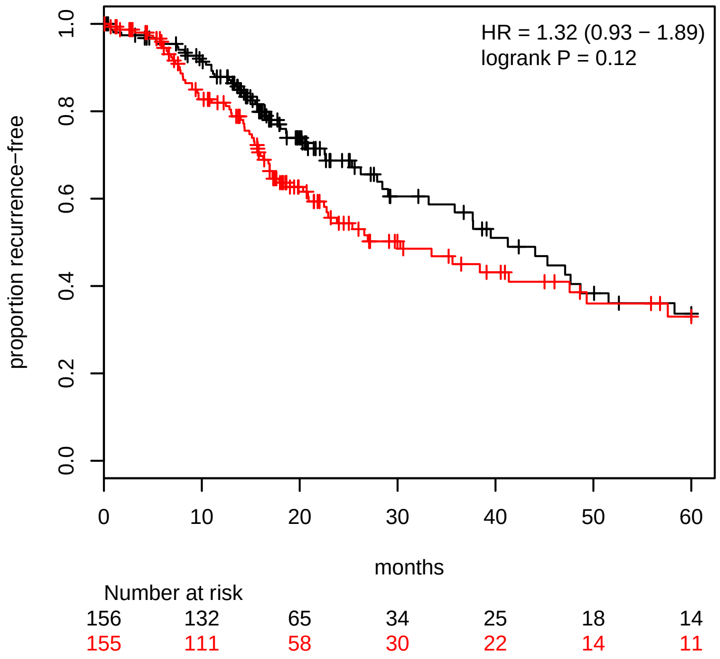

Supplementary Figure 1F
TCGA LUAD RNAseq − stage I and II
CCP, DFS − no treatment

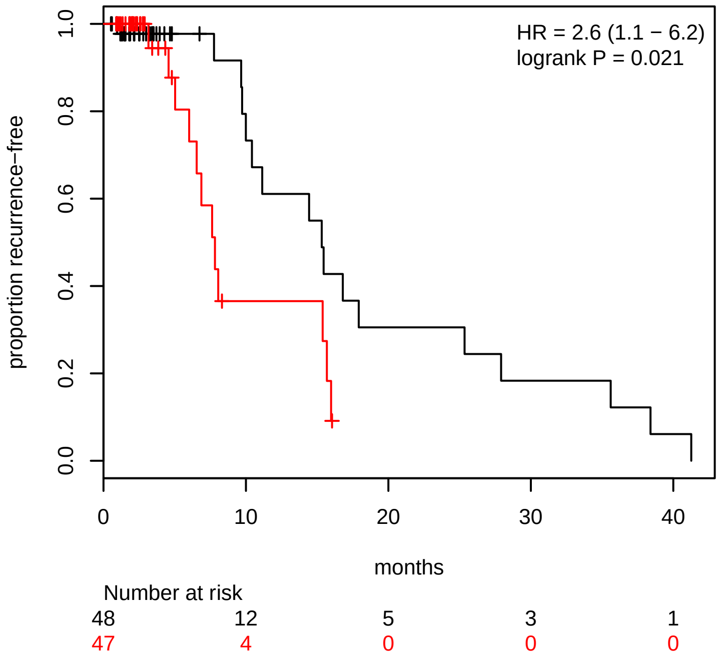

Supplement: Additional file 1: Figure S1A. — TCGA LUAD RNAseq − stage I and II ESLA − 7, DFS. Figure S1B: TCGA LUAD RNAseq − stage I and II ESLA − 7, DFS − no treatment. Figure S1C: TCGA LUAD RNAseq − stage I and II CIN25, DFS. Figure S1D: TCGA LUAD RNAseq − stage I and II CIN25, DFS − no treatment. Figure S1E: TCGA LUAD RNAseq − stage I and II CCP, DFS. Figure S1F: TCGA LUAD RNAseq − stage I and II CCP, DFS − no treatment. (PDF 20 kb) [file 40364_2016_58_MOESM1_ESM.pdf]
