# Supplementary material for: A robust prognostic gene expression signature for early stage lung adenocarcinoma
Source: Biomark Res. 2016 Feb 19;4:4. doi: 10.1186/s40364-016-0058-3 (PMC4761211; doi:10.1186/s40364-016-0058-3)
Supplement: Additional file 2: Table S1. — Performance of ESLA-7 in chosen lung squamous datasets. Median split according to ESLA-7 score. *p ≤ 0.05, **p ≤ 0.01, ***p ≤ 0.001. (DOCX 59 kb) [file 40364_2016_58_MOESM2_ESM.docx]

# Supplementary Tables

Supplementary Table 1. Performance of ESLA-7 in chosen lung squamous datasets. Median split according to ESLA-7 score. * - p ≤ 0.05, ** - p ≤ 0.01, *** - p ≤ 0.001.

| **Cohort** | **HR** | **CI** | **p** | **N** | **events** |
| --- | --- | --- | --- | --- | --- |
| Lee *et al.* 2008 | 1.5 | 0.7-3.1 |  | 61 | 30 |
| Zhu *et al.* 2010 | 2.1 | 0.6-7.2 |  | 26 | 11 |
| Rousseaux *et al.* 2013 | 1.1 | 0.5-2.5 |  | 57 | 22 |
| Botling *et al.* 2013 | 1.3 | 0.4-4.4 |  | 19 | 11 |
| TCGA LUSC 2013* | 0.53 | 0.3-1.0 |  | 115 | 43 |

* Data downloaded from the TCGA Data Portal using Data Matrix (https://tcga-data.nci.nih.gov/tcga/dataAccessMatrix.htm), hthgu133a platform, details and clinical information in file Supplementary_TCGA_LUSC_clinical_data.txt
