# Supplementary material for: A robust prognostic gene expression signature for early stage lung adenocarcinoma
Source: Biomark Res. 2016 Feb 19;4:4. doi: 10.1186/s40364-016-0058-3 (PMC4761211; doi:10.1186/s40364-016-0058-3)
Supplement: Additional file 4: Figure S2. — GSE8894 NSCLC Adeno untreated. (PDF 11 kb) [file 40364_2016_58_MOESM4_ESM.pdf]

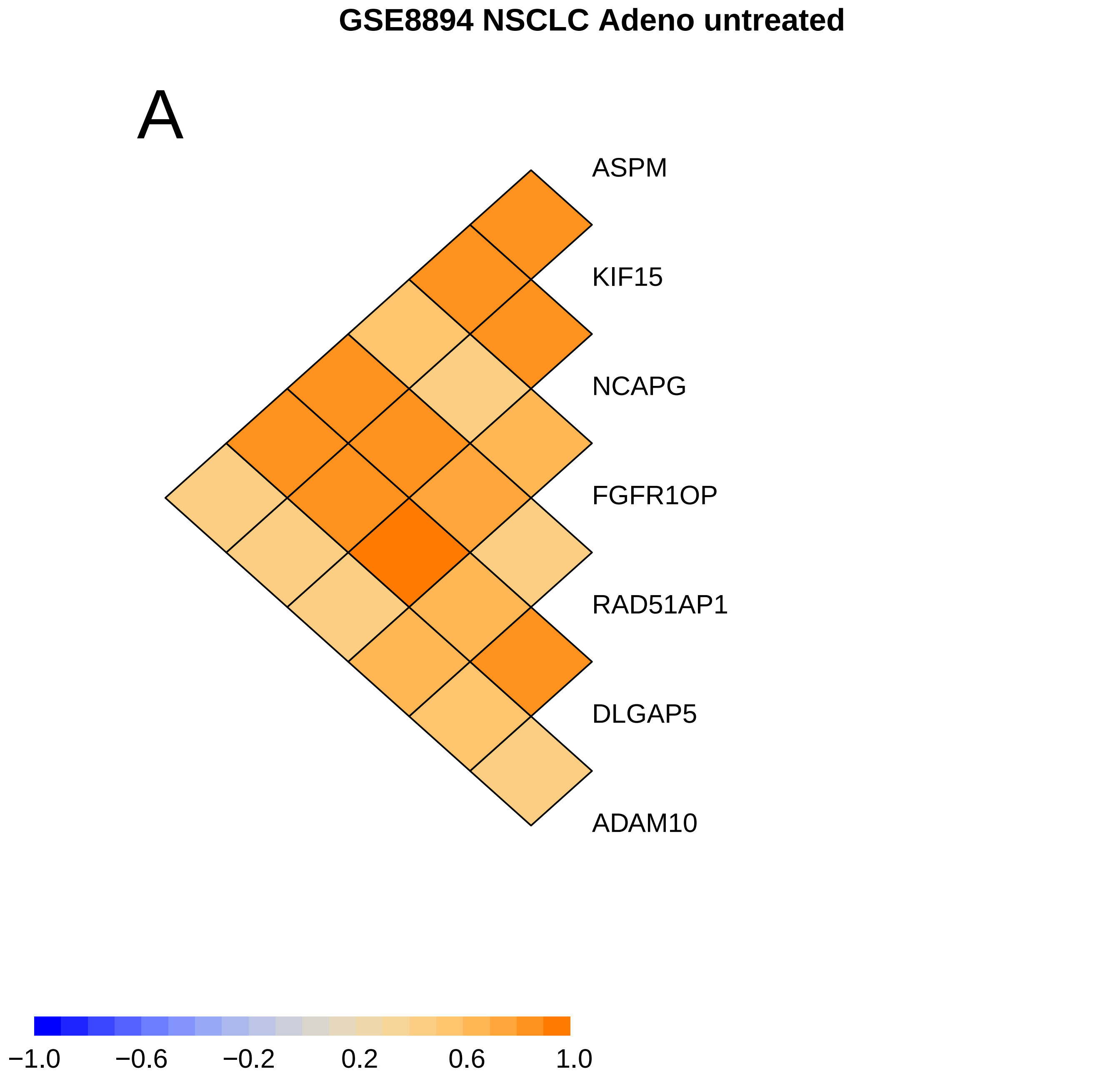
GSE8894 NSCLC Adeno untreated
A
ASPM
KIF15
NCAPG
FGFR1OP
RAD51AP1
DLGAP5
ADAM10
−1.0
−0.6
−0.2
0.2
0.6
1.0

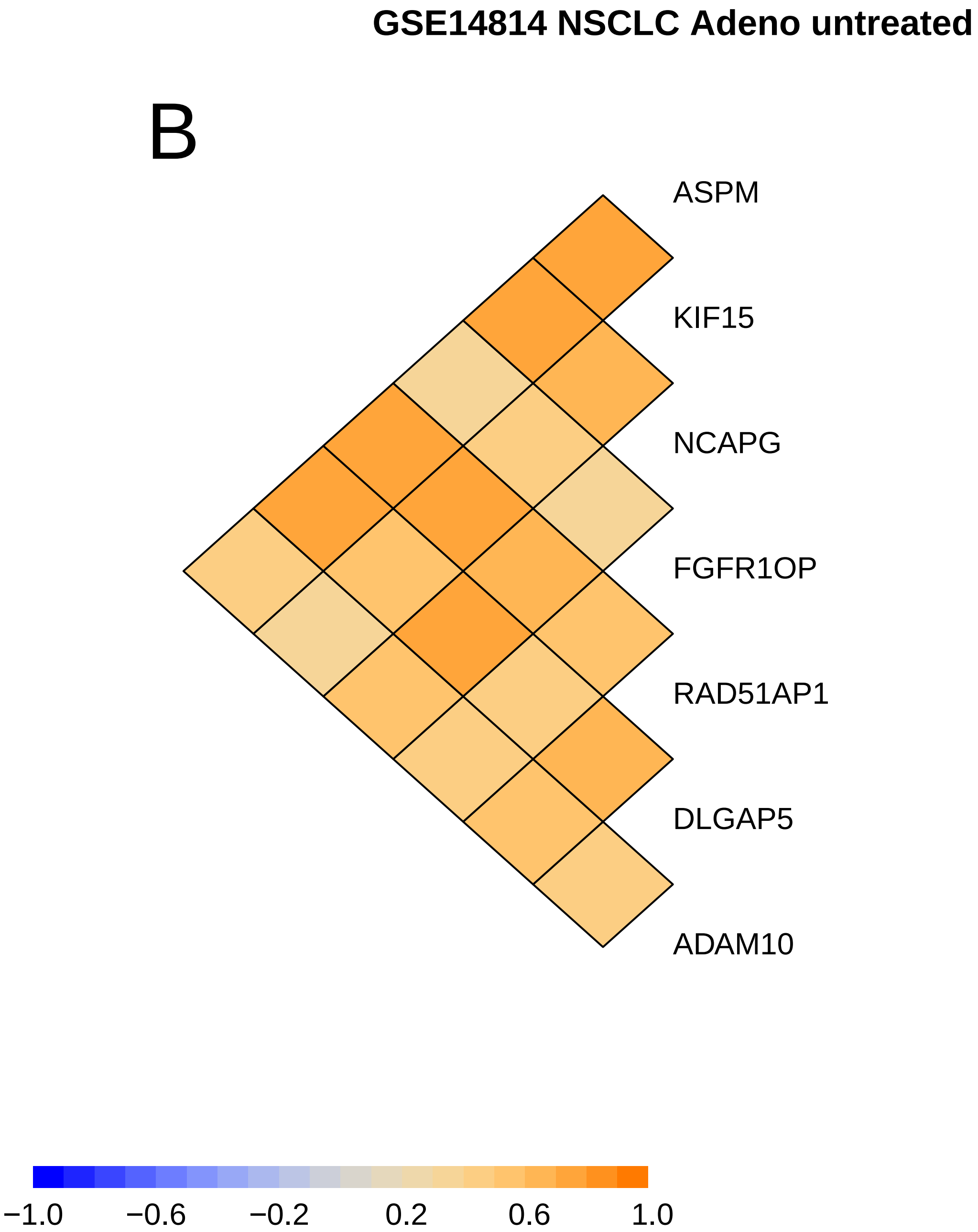

GSE14814 NSCLC Adeno untreated
B
ASPM
KIF15
NCAPG
FGFR1OP
RAD51AP1
DLGAP5
ADAM10
−1.0
−0.6
−0.2
0.2
0.6
1.0

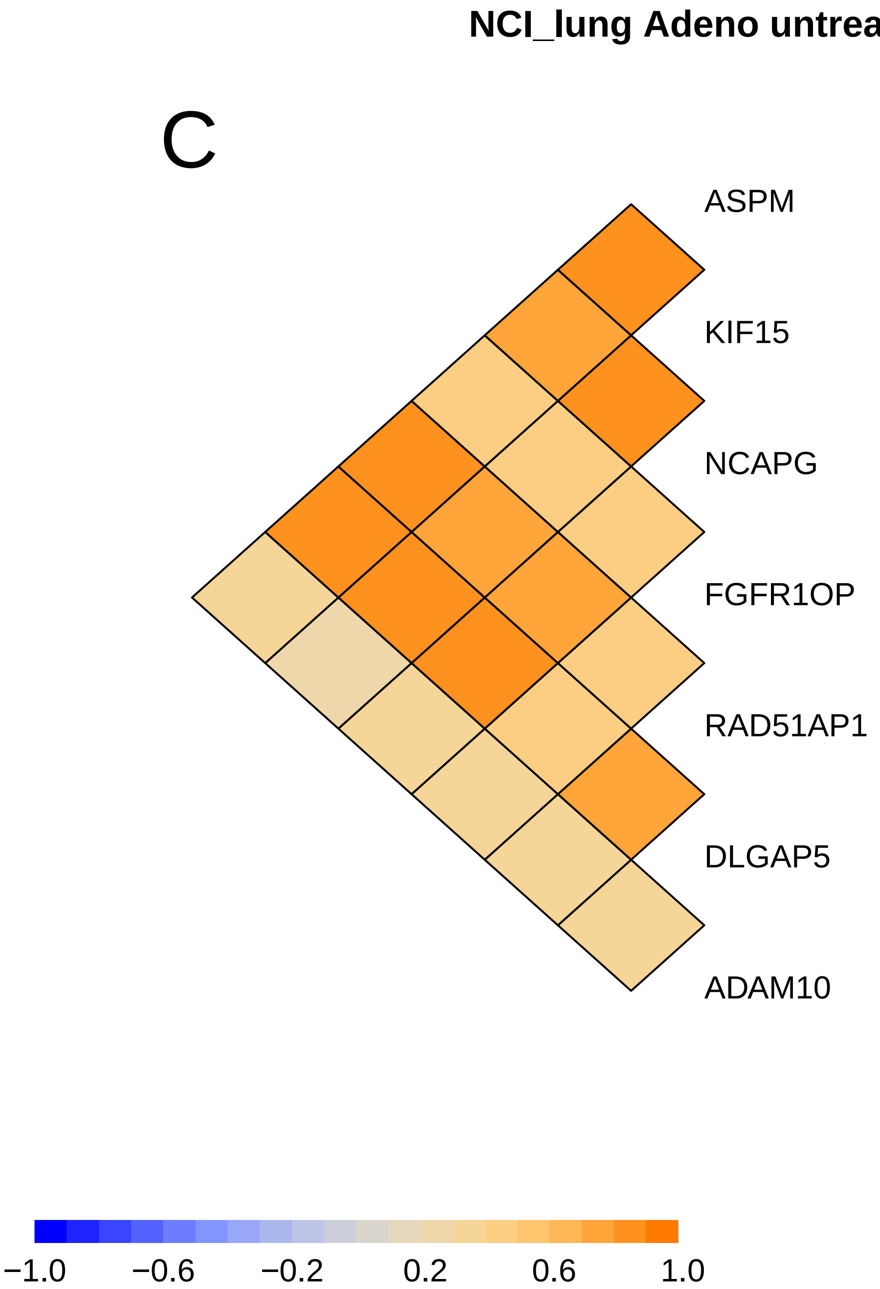
NCI_lung Adeno untreated
C
ASPM
KIF15
NCAPG
FGFR1OP
RAD51AP1
DLGAP5
ADAM10
−1.0
−0.6
−0.2
0.2
0.6
1.0

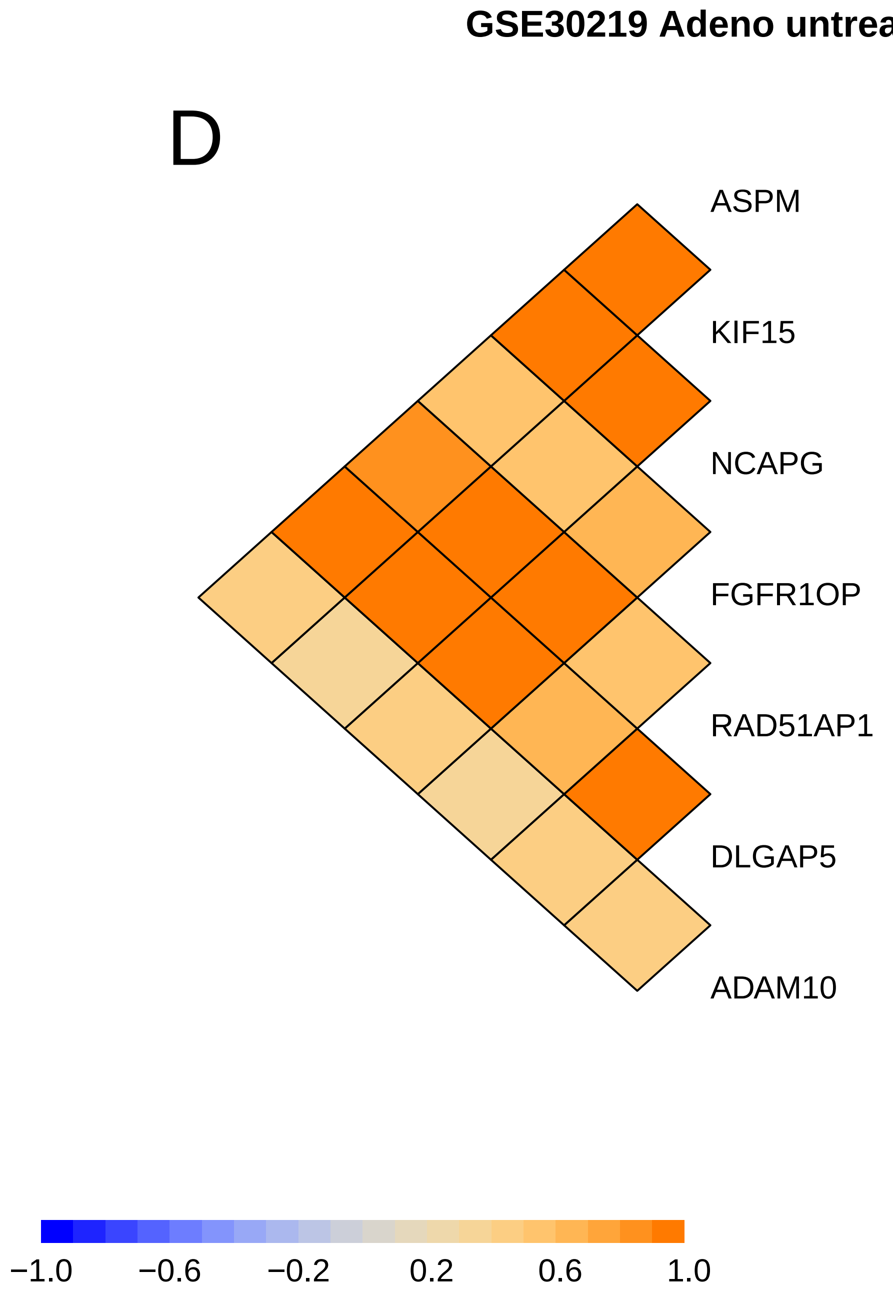
GSE30219 Adeno untreated
D
ASPM
KIF15
NCAPG
FGFR1OP
RAD51AP1
DLGAP5
ADAM10
−1.0
−0.6
−0.2
0.2
0.6
1.0

GSE31210 Adeno untreated

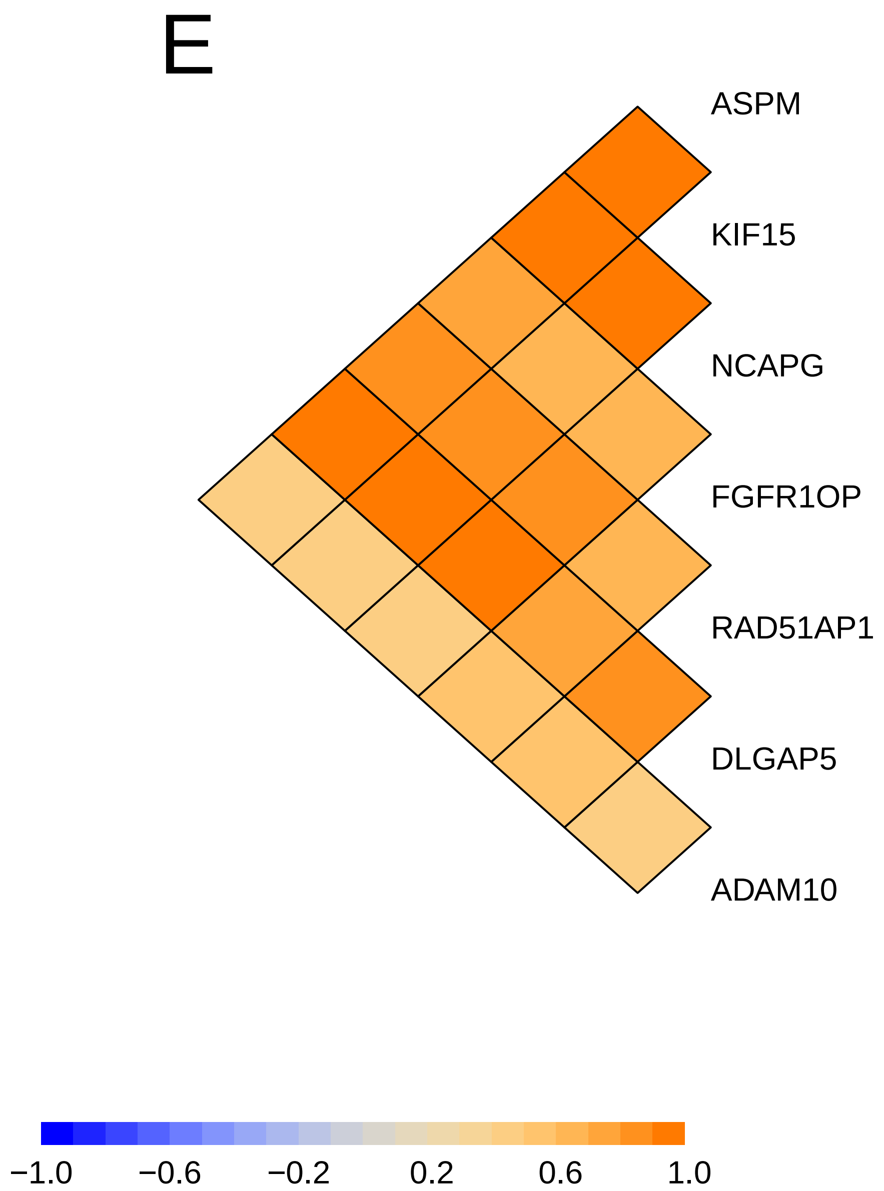

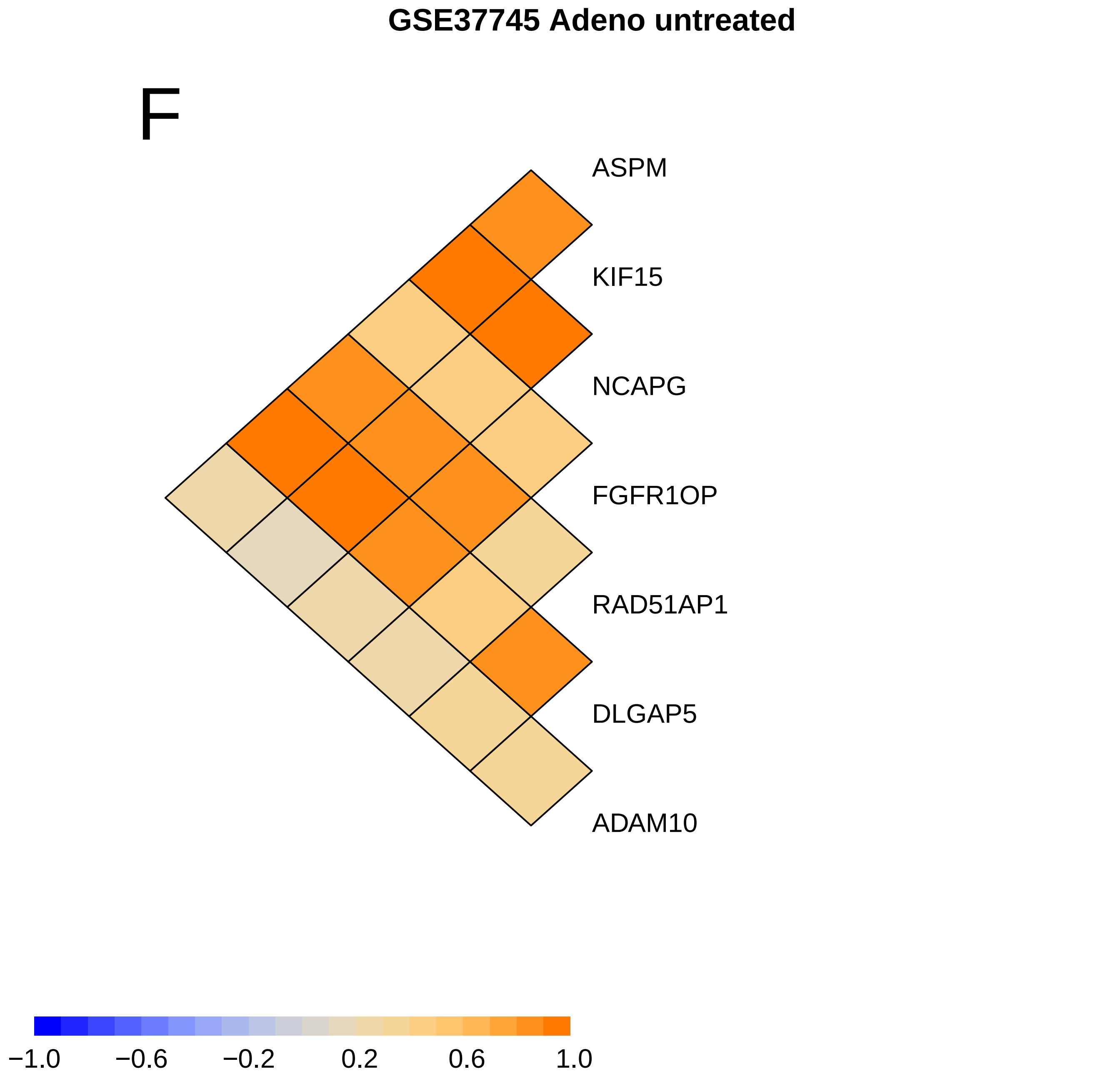

GSE37745 Adeno untreated
F
ASPM
KIF15
NCAPG
FGFR1OP
RAD51AP1
DLGAP5
ADAM10
−1.0
−0.6
−0.2
0.2
0.6
1.0

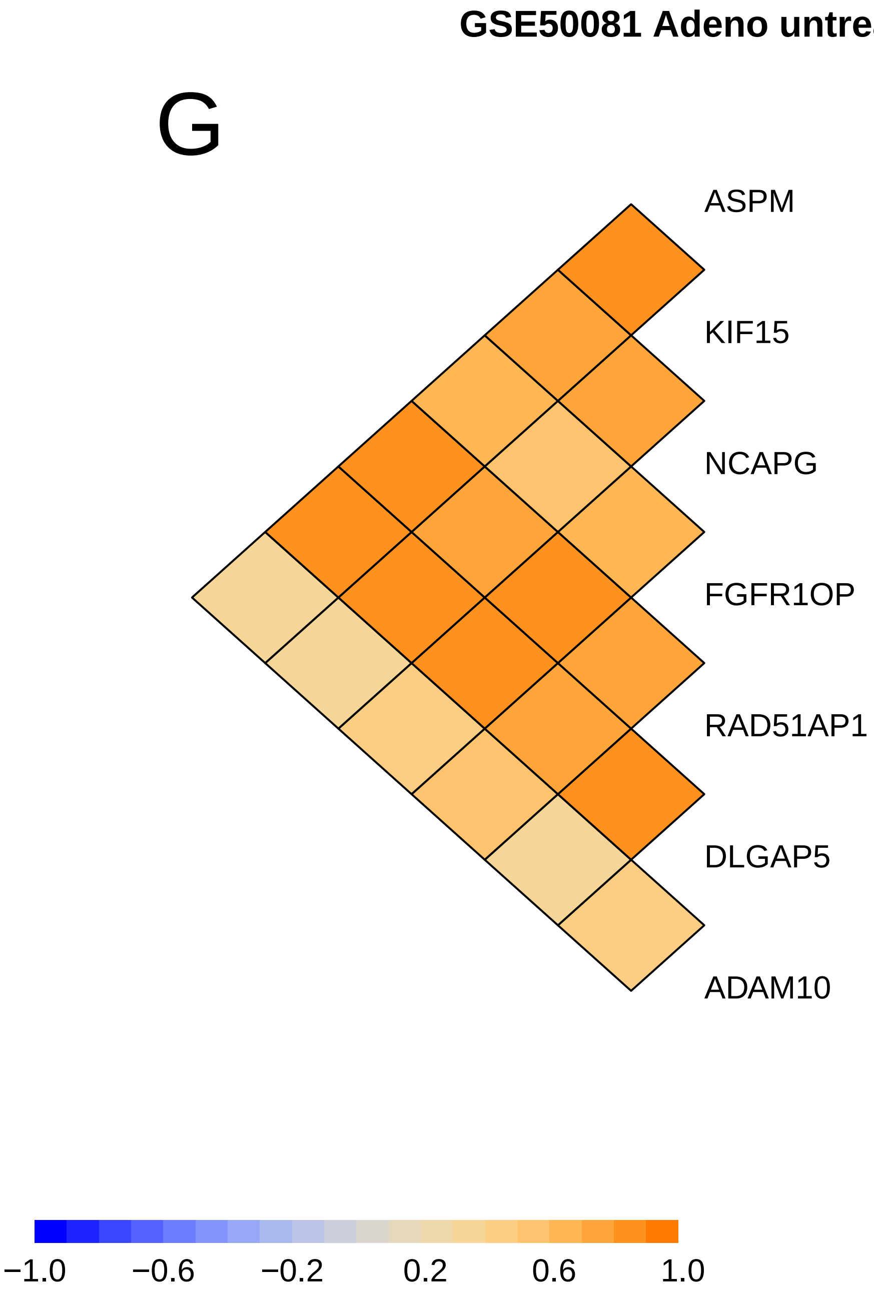
GSE50081 Adeno untreated
G
ASPM
KIF15
NCAPG
FGFR1OP
RAD51AP1
DLGAP5
ADAM10
−1.0
−0.6
−0.2
0.2
0.6
1.0
